# 纳米二氧化硅的肺毒性的研究进展

**DOI:** 10.3779/j.issn.1009-3419.2014.10.09

**Published:** 2014-10-20

**Authors:** 春 戴, 云超 黄, 永春 周

**Affiliations:** 650000 昆明，昆明医科大学第三附属医院（云南省肿瘤医院）胸心外科，云南省肺癌研究重点实验室 Department of Cardio-Thoracic Surgery, the Third Affiliated Hospital of Kunming Medical University (Yunnan Province Tumor Hospital), Yunnan Key Laboratory of Lung Cancer Research, Kunming 650000, China

**Keywords:** 纳米二氧化硅, 纳米颗粒, 肺毒性, 研究进展, Nanoparticles silicon dioxide, Nano-particle, Pulmonary toxicity, Research progress

## Abstract

纳米二氧化硅广泛分布于塑料、橡胶、陶瓷、涂料、粘合剂等众多领域，也是煤、石油等物质的燃烧的产物之一。越来越多的证据显示纳米二氧化硅与呼吸系统病变存在一定的相关性。本文综合国内外研究，对纳米颗粒的肺毒性进行概述，并从纳米二氧化硅的理化性质、暴露条件及其在肺部疾病的致病机制等方面进行综述。

## Abstract (en)

Nano-silicon dioxide widely distributed in plastic, rubber, ceramics, paint, adhesives, and many other fields, and it is the product of coal combustion. A growing evidence shows that nano-silicon dioxide has certain correlation with respiratory system disease. In this paper, we synthesized existing researches of domestic and abroad, summarized the lung toxicity of nanoparticles. This article are reviewed from the physical and chemical properties of nanoparticles silicon dioxide, exposure conditions and environment, and the pathogenic mechanism of nano-silicon dioxide.

随着纳米技术的飞速发展，纳米二氧化硅（nanoparticles silicon dioxide, nano-SiO_2_）作为纳米材料中的重要一员，其应用已扩展到疾病生物标记、生物运输载体、DNA运输载体等诸多领域。在日常生活中nano-SiO_2_也无处不在，其不但是新兴材料包括塑料、橡胶、陶瓷、涂料、粘合剂等的主要成分，也是煤、石油等矿物质燃烧的产物^[1]^。人类在nano-SiO_2_悬浮环境中的暴露可分为职业暴露和自然暴露。职业暴露包括陶瓷、橡胶、装潢等行业从业人员的暴露；自然暴露包括煤、石油燃烧的产物、空气污染等。nano-SiO_2_进入人体产生肺毒性主要经过呼吸道，晶体SiO_2_可导致矽肺，肺癌等肺部疾病，对肺部的损伤毋庸置疑^[2]^，但SiO_2_与肺部肿瘤的相关性尚不明确。在1997年，国际癌症研究机构（International Agency for Research on Cancer, IARC）把SiO_2_界定为人类的致癌因子^[3]^。但这个结论仍存在争议，因为当时对暴露在SiO_2_环境中的人群进行的研究未排除吸烟和其他致癌因子等干扰因素的影响。而在欧洲7个国家联合进行的INCO Copernicus study研究^[4]^提示人类暴露在SiO_2_环境中可明显提升肺癌的发病率，鉴于该项研究全面考虑了潜在的混杂因素，如吸烟和职业暴露，因此更具说服性。但由于SiO_2_与肺部肿瘤的相关性及其机制仍不明确，该领域成为近年来的研究热点。本文通过对纳米颗粒的概述及nano-SiO_2_的性状描述、环境分布和致癌机制等方面的综述，以期为nano-SiO_2_肺毒性的相关研究提供参考。

## 1 纳米颗粒的肺毒性与进入人体的途径

### 1.1 纳米颗粒的肺毒性概述

纳米颗粒是一种大小不超过100 nm的微型颗粒，在自然界中以乳胶体、聚合物、陶瓷颗粒、金属颗粒和碳颗粒等形式呈现。现今纳米颗粒正越来越多地应用于医学、防晒化妆品中，而环境中也悬浮大量的纳米颗粒物。由于粒径较小，纳米颗粒能够渗透到细胞中，并沿神经细胞突触、血管和淋巴血管传播。与此同时，纳米颗粒有选择性地积累在不同的细胞和亚细胞结构中。纳米颗粒的强渗透性不仅仅为药物的使用提供了可能性，同时，也对人体健康构成了威胁。2009年8月19日中国研究人员在“周三报告”中显示，在纳米涂料厂工作数月的多名中国年轻女性在没有适当保护措施的情况下罹患了永久性肺损伤，其中有两人死亡，这项研究首次记录了纳米技术对人类造成的健康损伤，检查发现，在这些女工的工作场所、支气管肺泡灌洗液、胸水和肺活检组织中均找到直径为30 nm的颗粒。Cha等^[5]^通过体外试验和大鼠的体内试验对纳米级锌（nano-Zn）、铁（nano-Fe）、硅（nano-Si）的细胞毒性进行检测，结果肯定了这三种纳米颗粒的细胞毒性。该实验采用的体外试验细胞分别是肝细胞（Huh-7）、脑细胞（A-172）、胃细胞（MKN-1）、肺细胞（A-549）和肾细胞（HEK293），纳米颗粒分别是Zn（300 nm）、Fe（100 nm）以及Si（10 nm-20 nm, 40 nm-50 nm, 90 nm-110 nm）。将这些细胞分别置入到纳米颗粒的环境中72 h后通过MTT比色法测量其细胞中线粒体的活性和DNA的含量，结果显示在不同纳米颗粒环境中线粒体活性与对照组相比均明显下降，同时伴随DNA含量出现0%-20%的下降，尤其在肝细胞和脑细胞中下降较胃细胞和肝细胞更明显，通过大鼠体内试验也得出了相同的结论。由此可见纳米颗粒对细胞的杀伤作用是客观存在的，但至今仍缺乏纳米颗粒对人体健康危害的充分研究，其具体致病过程尚未阐明。

### 1.2 纳米颗粒进入人体的途径

纳米颗粒存在于大气环境中，其主要通过以下途径进入人体产生损害。

#### 1.2.1 呼吸系统

呼吸道是纳米颗粒进入人体的主要途径^[6]^，纳米颗粒可以悬浮于空气当中，且其扩散速度与纳米颗粒的直径成反比，在空气中漂浮的时间越长，被机体吸入的机会也就越多。研究^[7]^发现纳米颗粒可在动物的呼吸道各部位及肺泡内沉积，虽然被吸入体内的纳米颗粒质量浓度并不高，但由于其粒径极小、数量大，从而为纳米颗粒致肺脏损伤提供了可能。

#### 1.2.2 消化系统

消化道也是纳米颗粒进入人体的重要途径之一，由于纳米材料在食品及药品中广泛应用，也为其通过消化系统进入人体提供了可能；此外，吸入的纳米颗粒也可通过人体的吞咽动作进入胃肠道。经消化道进入人体的纳米颗粒大部分可通过粪便排出体外，但少量的纳米颗粒可被消化道粘膜吸收，进入消化道毛细淋巴管，再引起胃肠道粘膜细胞的免疫应答反应，同时其也通过粘膜下层进入毛细血管网并到达全身各组织器官形成再次分布^[8]^。

#### 1.2.3 皮肤

皮肤是人体阻挡外界的物理和化学刺激的重要屏障系统，对绝大部分损伤物质具有初始隔离作用，但纳米颗粒由于粒径微小，经简单扩散或渗透等形式既可通过皮肤角质层进入皮下组织进而被人体吸收^[9]^。大气及生产环境中的纳米颗粒是与皮肤接触的主要途径，而纳米材料在化妆品、纺织品及药物等领域的应用同样增加了纳米颗粒通过皮肤进入人体的机会。

#### 1.2.4 血液系统

纳米材料在医疗领域的应用，也为其提供了进入人体新的途径。如医学影像技术、药物的缓释控释、靶向载药、肿瘤的诊断及治疗等方面的应用，都增加了纳米颗粒通过静脉血液系统进入人体的机会^[10]^。由于纳米材料粒径较小、理化性质特殊，进入人体后不易控制等原因，故此种途径进入人体后其危险性不容忽视。

由此可知，纳米颗粒包括nano-SiO_2_主要通过呼吸道、消化道、皮肤进入机体，并可以通过各种途径进入血液循环和淋巴循环而后分布到全身，到达潜在的敏感靶位点，进而产生一系列致病作用。这些也成为近年对纳米颗粒肺毒性的研究热点和防治重点。

## 2 nano-SiO_2_的性质及其暴露的环境

### 2.1 nano-SiO_2_的性质

nano-SiO_2_是一种直径介于1 nm-100 nm之间的SiO_2_粒子，因早期研究认为其对生命体不会造成直接的危害而被认为是安全的纳米生物材料，已被广泛用于疾病诊断、生物分析和成像、药物载体等研究中^[11]^。由于nano-SiO_2_的性质与其他材料不同，从而导致它具有不同的物理和化学特性。nano-SiO_2_有很高的表面积，该特性能促进细胞对nano-SiO_2_的吸收，从而对肺、大脑、骨髓、淋巴结和心脏产生细胞、亚细胞、蛋白质和基因水平的损害。由于这些潜在的毒性作用，nano-SiO_2_对人类健康的影响正是一个新兴的研究领域。

### 2.2 nano-SiO_2_的暴露环境

日常生活中nano-SiO_2_存在于油漆、涂料、塑料、合成橡胶、粘合剂及密封剂中，亦存在于煤、石油等矿物质的燃烧产物中。男性从事砖瓦工、木匠和建筑工人等职业都暴露于SiO_2_粉尘，这些职业暴露因素都增加了肺癌的发病风险，且这些因素和吸烟对肺癌的影响是独立的。一项欧洲的多中心研究（multicentre case-referent study）发现，综合考虑了性别、年龄等因素后暴露于SiO_2_粉尘中的非吸烟者的癌症发病率显著提高^[12]^。1979年-2006年印度的肺癌流行病学研究也显示：暴露在二氧化硅环境中的患者肺癌的发病率接近普通未暴露在SiO_2_环境中患者肺癌发病率的两倍^[13]^。而在国内，中国云南省宣威地区是世界非吸烟女性肺癌发病率最高的地区之一，既往研究^[14, 15]^表明，宣威地区女性肺癌高发与当地开采和使用的C1烟煤及其燃烧产物（底灰和烟尘）中含有的大量超细SiO_2_颗粒物紧密联系，目前该课题组正在深入探讨其作用机制。

由此可见nano-SiO_2_不仅存在于生物材料中，其也在自然界中广泛分布。所以人体不仅在职业生产过程中会暴露于SiO_2_环境中，其在日常生活中也会暴露于SiO_2_环境中，加之nano-SiO_2_颗粒直径小，可悬浮在空气中，从而增加了人体的暴露风险。以上实验也同时证明了nano-SiO_2_的肺毒性。

## 3 nano-SiO_2_致病机理的研究进展

目前对nano-SiO_2_的肺毒性的致病机理的研究主要集中在细胞水平和基因水平。细胞水平主要是破坏细胞膜、损伤细胞器及改变细胞的生长周期而基因水平的损伤则主要通过氧化应激系统（reactive oxygen species, ROS）损伤遗传物质。

### 3.1 细胞水平危害的相关研究

纳米级SiO_2_很容易进入细胞膜，并在细胞质中积累，从而破坏细胞的新陈代谢、诱导细胞功能障碍、癌变、甚至死亡^[16-18]^。nano-SiO_2_对细胞的损害已得到大量实验的证实，而nano-SiO_2_的直径是否与其损伤程度成相关性，不同的学者得到了不同的结论。

Kasper等^[19]^通过检测细胞毒性因子以及炎症反应因子来测定不同大小nano-SiO_2_（30 nm, 70 nm, 300 nm）对肺上皮细胞NCI H441细胞株和内皮细胞的ISO-HAS-1细胞的毒性作用，研究显示flotillin蛋白介导了细胞对nano-SiO_2_的吞噬。在该项研究中，去除flotillin蛋白的H441细胞对不定形nano-SiO_2_的吸收明显减少，与对照组相比，去除flotillin蛋白的细胞暴露在nano-SiO_2_环境中的生存率也明显下降；同时该研究也证实了纳米颗粒大小对细胞毒性和炎症反应的负相关关系，即纳米颗粒的直径越小对细胞造成有害影响就越大。

Duan等^[20]^通过实验及形态学检查发现，经过15 nm、30 nm、100 nm和微米级SiO_2_粒子10 g/mL接触24 h的HaCaT细胞的生长明显被抑制，细胞失去细胞壁变得萎缩，甚至死亡，被15 nm和30 nm SiO_2_浸润的细胞比微粒SiO_2_浸润的细胞形态的变化更明显。此外，有研究^[21]^也显示：SiO_2_诱导产生的ROS可以通过脂质过氧化反应引起细胞膜损伤。值得一提的是，有研究测定了暴露在无定形纳米（10 nm、80 nm）SiO_2_中48 h的A549细胞的细胞毒性（通过MMT和LDH测定）和氧化应激反应（ROS水平，膜脂质过氧化、谷胱甘肽水平和活动的谷胱甘肽代谢酶），结果提示：nano-SiO_2_主要通过ROS和膜脂质过氧化作用对细胞产生损伤，而不是消耗谷胱甘肽（glutathione, GSH）^[22]^。也有相反的研究不支持nano-SiO_2_粒径与肺损伤呈负相关关系，如Cha等^[5]^用MTT比色法测量了暴露在nano-SiO_2_环境中肺细胞的活性，但结果提示10 nm-20 nm、40 nm-50 nm、90 nm-110 nm的SiO_2_均可使肺细胞发生不同程度的损伤，而损伤程度与nano-SiO_2_的直径无直接关系。

综上所述，nano-SiO_2_对机体的毒作用机制有氧化损伤及炎症反应、影响细胞凋亡改变细胞周期、细胞膜毒性以及DNA损伤等多个方面。虽然国内外已有相关研究，但对nano-SiO_2_颗粒如何进入线粒体并导致线粒体损伤的机理，以及等剂量同浓度的不同颗粒直径的纳米颗粒对机体毒性作用等方面还有待深入研究。

### 3.2 基因水平危害的相关研究

有研究^[23]^显示nano-SiO_2_可对细胞造成损伤，影响细胞复制、转录、增殖，在一定条件下可以转移到细胞核内，也可造成主要靶器官肺脏及脾脏的损伤。nano-SiO_2_的基因毒性主要由炎症反应中的ROS途径介导，加之染色质结构的变异、溶酶体释放脱氧核糖核酸酶，从而产生了损伤细胞遗传物质稳定性的复杂诱导物^[24]^。

Musa等^[25]^通过彗星实验和小鼠淋巴瘤细胞实验（mouse lymphoma assay, MLA）检测了小鼠的淋巴结细胞和人肺支气管上皮细胞（BEAS-2B）在nano-SiO_2_环境下的基因毒性，结果提示nano-SiO_2_能够引起细胞毒性和初级DNA损伤而不会导致染色体水平的删除及变异Gerloff等^[26]^将结肠腺癌细胞（Caco-2细胞模型）暴露在nano-SiO_2_中24 h后可引起DNA的损伤和总GSH的耗竭，并最终导致细胞的死亡。

Cha等^[5]^测量暴露在nano-SiO_2_环境中细胞核DNA的含量（将具有荧光性的双苯酰亚胺H33258植入到染色体中从而测量DNA的含量），其结果提示10 nm-20 nm、40 nm-50 nm、90 nm-110 nm的SiO_2_均可使DNA发生改变。

以上研究提示：体积小和高表面积的nano-SiO_2_能导致难以预测的基因毒性，人体的氧化应激系统参与了nano-SiO_2_对人体遗传物质的损伤，而且不同粒径的nano-SiO_2_对DNA的损伤程度存在差异，而在细胞代谢、信号通路及与其他物质伴随作用等方面的机制仍具有广泛的研究前景。

## 4 nano-SiO_2_的肺毒性的研究总结及展望

nano-SiO_2_广泛存在于油漆、涂料、塑料、合成橡胶、粘合剂及密封剂中，亦存在于煤、石油等矿物质的燃烧产物中，可通过呼吸系统、消化系统和血液系统等途径对人体产生损伤，其与肺癌的相关性已经得到肯定^[27]^，但具体致癌机制尚不清楚。如：Kasper等^[19]^对flotillin蛋白介导nano-SiO_2_的内吞作用提出了假设，但未对其具体过程进行论证。Cha等^[5]^通过对染色体的测定也观察到nano-SiO_2_对基因有明显的损伤，但也未阐明其损伤机制。这些都为我们研究nano-SiO_2_对人体呼吸系统的损害机制提供指导和切入点。
